# Lung cancer screening in Appalachian Kentucky: The impact of Lung-RADS on subsequent testing and cancer identification

**DOI:** 10.1017/cts.2019.416

**Published:** 2019-09-12

**Authors:** Roberto Cardarelli, Vashisht Madabhushi, Kacie Bledsoe, Anthony Weaver

**Affiliations:** 1 Department of Family & Community Medicine, University of Kentucky, College of Medicine, 2195 Harrodsburg Road, Lexington, KY, 40504-3504 USA; 2 Department of General Surgery, University of Kentucky, College of Medicine, 800 Rose Street, Lexington, KY, 40536 USA; 3 St. Claire Regional Medical Center, 222 Medical Circle, Morehead, KY 40351 USA

**Keywords:** Lung cancer screening, Rural health, NLST, Lung cancer, Lung-RADS

## Abstract

The National Lung Cancer Screening Trial (NLST) demonstrated the use of low dose helical computed tomography (LDCT) scans for lung cancer screening. However, the NLST was implemented in urban hospitals and prior to the Lung CT Screening Reporting and Data System (Lung-RADS). In this retrospective cohort study, 774 eligible patients received LDCT screening using Lung-RADS criteria. Eighty-four patients (10.9%) had subsequent testing performed compared to 24.2% in the NLST study. Of those with subsequent testing, 21.4% were diagnosed with lung cancer compared to only 4.6% in the NLST study. Lung-RADS significantly reduced unnecessary testing while identifying higher rates of lung cancer compared to the NLST.

## Introduction

Lung cancer is the leading cause of cancer-related mortality in the USA, accounting for one in four cancer-related mortalities [1]. Kentucky has the highest incidence of lung cancer in the country at 91.4 per 100,000 compared to the national rate of 58.3 per 100,000 [2]. Furthermore, Appalachian Kentucky counties have an even higher incidence rate of lung cancer, at 107.2 per 100,000. According to the Appalachian Regional Commission, the majority of Kentucky’s Appalachian counties are under significant economic distress, which has been associated with overall poor health [3,4]. The Appalachian region has been identified as a medically underserved region due to the financial, geographic, and health system challenges in the region [5].

While there have been tremendous strides in screening for other types of cancers, such as colon, prostate, and cervical, lung cancer screening still remains an area of controversy. The National Lung Cancer Screening Trial (NLST) was a large, multicenter, randomized controlled trial that compared annual low dose helical computed tomography (LDCT) to chest X-ray in high-risk individuals as a tool for lung cancer screening [6,7]. The NLST demonstrated a statistically significant improvement in survival with annual LDCT in individuals who are high risk for lung cancer. However, because site selection for the study was among 33 urban or metropolitan institutions across the country, the feasibility of implementing a screening program similar to NLST in a rural population, similar to the population present in Appalachian Kentucky, is unknown. Moreover, the positive predictive value of diagnostic and screening tests is dependent on the disease incidence of the population in which it is used [8]. Thus, the impact of LDCT screening in a region with high rates of lung cancer may be underrepresented.

Additionally, the NLST was conducted prior to the advent of Lung CT Screening Reporting and Data System (Lung-RADS) [9]. Lung-RADS is an effort by the American College of Radiology (ACR) to standardize reporting of the screening LDCT results based on lung cancer risk. Lung-RADS categorizes the imaging finding of a screening LDCT from 0 to 4. As the number increases, the likelihood of malignancy is higher. Based on categorization of the imaging findings, the Lung-RADS guidelines recommend additional imaging and diagnostic testing to be performed to confirm the diagnosis [9]. Retrospective application of the Lung-RADS criteria to the NLST results showed not only a potential reduction in the false-positive rate, but also a decrease in the sensitivity [10]. This may lead in fewer subsequent testing, associated risks, and reduce anxiety and fear among patients. The purpose of this study was to evaluate the distribution of LDCT findings by Lung-RADS categories in a rural Appalachia community hospital, determine the number of subsequent testing after baseline LDCTs, and the number of cancers identified and to compare these results to NLST baseline data.

## Methods

### Lung Cancer Screening Program

St. Claire Healthcare (SCR) is a 159-bed community hospital in NE Kentucky and is registered with ACR to perform lung cancer screening. SCR provides healthcare services to 11 counties in northeastern Kentucky, all of which are rural counties, with a total population of 166,130 people served. Their lung cancer screening program commenced in 2015 and includes trained on-site radiologists who read all LDCTs and a data manager to ensure all data are entered into the ACR system. Annual reminders are sent out to patients by the radiology suite but shared decision-making and tobacco cessation must be performed and documented by the ordering provider, which is verified by radiology. All readings follow the ACR Lung-RADS reporting criteria and data are uploaded into the SCR ACR database for tracking and monitoring purposes.

### Data Collection and Outcome Measures

SCR de-identified medical record and ACR data from August 2015 to August 2018 were used to determine the distribution of findings by Lung-RADS category. Details of the Lung-RADS categorization can be found on the ACR website at: https://www.acr.org/Clinical-Resources/Reporting-and-Data-Systems/Lung-Rads. While the Lung-RADS criteria were not in effect during the NLST study, the retrospective analyses of the NLST study by Lung-RADS categories by Pinsky *et al*. were used to compare SCR results [10]. In that study, the Lung-RADS categories were collapsed according to management recommendations and probability of malignancy, which resulted in three categories: 1/2, 3, 4A/4B/4X. To compare our results to the Pinsky *et al*. results, we also collapsed the Lung-RADS results from our study into the same categories. Some readings in the NLST had more than one category findings (example 3 and 4B). For these cases, we attributed their count to the category with the greatest probability of malignancy.

We further assessed the number of subsequent testing till diagnosis (if any), ranging from additional imaging to resections performed based on the findings of the LDCT, and calculated the percentage that resulted in a cancer diagnosis. SCR results were then compared to NLST baseline LDCT results and number of subsequent testing and cancer diagnoses. Subsequent testing in our study included follow-up imaging, biopsies, or any other procedures and the definition was mirrored as it was described in the NLST study. All study procedures were approved by the University of Kentucky and St. Claire Regional Medical Center Institutional Review Boards.

## Results

There were a total of 774 SCR patients who received a baseline LDCT for lung cancer screening between August of 2015 and August of 2018 (Table 1). The mean age at the time of the LDCT was 63 ± 6 years. The patients were 49.9% female and 91.2% were White, which represents the general Appalachia population. The mean pack years smoked was 54, with a standard deviation of 29 pack years. Sixty-nine percent of patients were current smokers and the remainder were former smokers who quit less than or equal to 15 years ago.

**Table 1.** Study population (N = 774)

Characteristics	Years	Standard deviation
Mean age	63 years	6 years
Mean pack years smoking	54 years	29 years
	**N**	**%**
Current smokers	534	69.0%
Gender		
Male	388	50.1%
Female	366	49.9%
Race		
White	706	91.2%
Non-White	68	8.8%

Among baseline SCR LDCT readings, 85.2% were categorized as Lung-RADS 1 or 2, 8.0% as Lung-RADS 3, and 6.7% as Lung-RADS 4A, 4B, or 4X (Table 2). These rates are comparable to the NLST Lung-RADS categorization rates found in Pinsky *et al*.’s retrospective analyses of the NLST trial [10].

**Table 2.** St. Claire Healthcare (SCR) findings vs. The National Lung Cancer Screening Trial (NLST) [7]

Lung-RADS category	Baseline LDCT, SCR (*n* = 774)	%	Baseline LDCT, NLST (*n* = 26,455)	%
1/2*	659	85.2%	22,854	86.4
3	62	8.0%	1697	6.4
4A/4B/4X	52	6.7%	1904	7.2

LDCT, low dose helical computed tomography; Lung-RADS, Lung CT Screening Reporting and Data System.

In terms of subsequent testing and cancer diagnoses, we found 84 patients (10.9%) had further testing performed at SCR compared to 24.2% in the NLST study (Table 3). Majority of those who had subsequent testing had non-invasive testing. Eighteen (2.3%) of the 774 SCR patients screened were ultimately diagnosed with a lung malignancy compared to only 1.1% in the NLST study. More importantly, among SCR patients who had subsequent testing (*n* = 84), 21.4% were diagnosed with cancer compared to 4.6% in the NLST study who had subsequent testing.

**Table 3.** Screening low dose helical computed tomography scans (LDCTs), subsequent testing, and lung cancers identified in St. Claire Healthcare (SCR) vs. The National Lung Cancer Screening Trial (NLST)

	SCR data (n)	%	NLST data (n)	%
Screening LDCTs	774	–	26,309	–
Additional testing obtained	84	10.9	6369	24.2
Cancers identified among screened	18	2.3	270	1.1
Cancers identified as % of those with additional testing	18	21.4	270	4.2
Patients with additional testing	84	–		
Only one additional test	56	66.67		
Two additional tests	25	29.76		
Three additional tests	3	3.57		
Total additional tests performed	115	–		
CT scans	60	52.17		
PET CTs	35	30.43		
Biopsies	15	13.04		
Surgical procedures	4	3.48		
Other	1	0.87		

## Discussion

Controversy surrounded the announcement of the lung screening guideline when published by the United State Preventive Services Task Force [11]. The concern for high false-positive rates and having a guideline based on evidence from one large multicenter study led to a Medicare Evidence Development & Coverage Advisory Committee recommendation to not cover the testing by the Centers for Medicare and Medicaid Services and for one of the professional organizations not to endorse the guideline (the American Academy of Family Physicians) [12,13]. These actions were based on data prior to the systematic application of the Lung-RADS when reading and recommending follow-up of LDCT findings. Moreover, based on current guidelines, tobacco cessation counseling and shared decision-making is required as part of the lung cancer screening process.

In this study, we are able to demonstrate how a small rural community hospital successfully screens lung cancer in a region with one of the highest lung cancer rates in the USA. The utilization of Lung-RADS in the screening protocol demonstrated 13.3% fewer additional testing performed, when compared to the NLST. Among SCR patients who had additional testing performed, the percentage of patients identified with lung cancer was approximately five times higher compared to the NLST population. This demonstrates that the application of Lung-RADS in the screening procedures resulted in fewer subsequent testing procedures despite the high rate of lung cancer identified compared to the NLST. The rates of cancer identified at SCR can also be attributable to the fact that the prevalence of lung cancer is highest in Eastern Kentucky compared to the rest of the USA. This is an important factor when determining the value of LDCT screening in populations with high risk of developing lung cancer. Overall, lung cancers identified through lung cancer screening (LCS) in Eastern Kentucky were double (2.3%) compared to the NLST study (1.1%) [6]. We also found higher rates of lung cancer from screening compared to a multisite implementation study in the Veterans Administration (VA) system. This VA study identified 56.2% of the patients screened needed additional tracking, while lung cancer was eventually found in only 1.5% of the study population [14].

The NLST was conducted across urban and metropolitan sites. The infrastructure, resources, and access are different in rural communities. A secondary aim of this study was to evaluate the feasibility of implementing LDCT screening guidelines in a rural community. We sought to assess whether subsequent testing rates would be higher compared to the NLST study. In fact, SCR conducted fewer subsequent tests compared to both the NLST study and VA system study [7,10,14]. Since tracking of results and repeat testing over time drives fear, anxiety, and concern among patients, the importance of reducing unnecessary testing cannot be emphasized enough. When we compared the number of cancers identified among those who had subsequent testing after their baseline LCS, SCR identified more cancers (21.4%) compared to those with subsequent testing in the NLST study (4.6%). To our knowledge, this is the first study to demonstrate that.

There are limitations in our study that should be noted. While we compared SCR screening outcomes to a study that applied the Lung-RADS criteria post-hoc to the NLST study, there could be significant differences in false-positive rates and subsequent testing rates if the Lung-RADS was actually applied during the NLST a priori. Nonetheless, the intention of our study was to demonstrate the difference of subsequent testing rates with the application of Lung-RADS criteria at the time of LCS compared to the NLST study, which lacked such criteria. Another limitation is that we conducted the study in a region of the country with the highest rates of lung cancer [2]. This impacts the positive and negative predictive value of LDCT in screening for lung cancer [8]. Conversely, this finding has important policy and health system implications when the value of implementing a LCS program is being considered. This is especially important in rural areas where resource allocation is a critical decision factor as overall resources are limited. Finally, we cannot generalize our findings beyond that of SCR, as LCS programs can have significant variability. This was also found in closed systems with multiple sites, such as the VA system [14]. Hence, further studies must be replicated in other rural regions in the USA with varying incidence rates of lung cancer.

We conclude that the application of the Lung-RADS has the potential to reduce unnecessary testing; and when testing is warranted, a higher rate of lung cancers can be identified. Further research is needed to assess whether these findings are replicable in other rural regions where lung cancer is comparable to national rates.
